# The Efficiency of Barley (*Hordeum vulgare*) Bran in Ameliorating Blood and Treating Fatty Heart and Liver of Male Rats

**DOI:** 10.1155/2015/740716

**Published:** 2015-03-17

**Authors:** Khalid O. Abulnaja, Haddad A. El Rabey

**Affiliations:** ^1^Biochemistry Department, Faculty of Science, King Abdulaziz University, Jeddah 21589, Saudi Arabia; ^2^Genetic Engineering and Biotechnology Institute, Sadat City University, P.O. Box 79, Sadat City, Minufiya, Egypt

## Abstract

The current study focused on testing the hypolipidemic activity of two doses of barley bran on hypercholesterolemic male rats. Twenty-four male albino rats weighing 180–200 gm were divided into four groups. The first group (G1) was the negative control, the second group (G2) was the positive control group fed 2% cholesterol in the diet, and rats of the third and the fourth groups were fed 2% cholesterol and were cosupplemented with 5% and 10% barley bran, respectively, for 8 weeks. The hypercholesterolemic rats of (G2) showed an increase in lipid profile, liver enzymes, lactate dehydrogenase, creatine kinase-MB, and lipid peroxide and a decrease in antioxidant enzymes, whereas kidney function, fasting blood sugar, glycated hemoglobin total protein, and total bilirubin were not significantly affected compared with the negative control group in G1. Moreover, histology of heart, liver, and kidney of G2 rats showed histopathological changes compared with the negative control. Administration of the two doses of barley bran in G3 and G4 to the hypercholesterolemic rats ameliorated the level of lipids, liver enzymes, lactate dehydrogenase, and creatine kinase-MB. In addition, the histology of heart, liver, and kidney tissues nearly restored the normal state as in G1.

## 1. Introduction

Increase in blood cholesterol and lipoproteins causes lipid deposition in cardiovascular tissues with reduced HDL cholesterol leading to uncontrolled dyslipidemia and heart disease [1]. Several factors in obesity including hyperlipidemia, hyperinsulinemia, hyperleptinemia, and insulin resistance could contribute to the development of type 2 diabetes [2, 3]. Obesity has been associated with increased triglyceride, VLDL, total cholesterol, and decreased HDL and, thus, is also a cause of CVD [4]. Obesity is closely related to diabetes and responsible for increased number of diabetes in recent years [5, 6]. Complementary and alternative medicine is emerging in prevention of chronic coronary and heart diseases as safe practice because of the high risk of mortality and long-term morbidity associated with surgical procedures of CAD and high side effects of chemotherapy [7].

Whole-grain cereals protect the body against age-related diseases such as diabetes, cardiovascular diseases, and some cancers [8]. Barley bran contains *β*-glucans (beta-glucans) which is polysaccharides of D-glucose monomers linked by *β*-glycosidic bonds. *β*-Glucans are a diverse group of molecules that can vary with respect to molecular mass, solubility, viscosity, and three-dimensional configuration [9]. The administration of barley bran may help to reduce appetite and weight gain and ameliorate lipid profile [10, 11]. The viscosity determined by water solubility and molecular weight has been shown to affect the hypocholesterolemic effect of beta-glucans [12]. Hull-less barley brans consist of mannose, galactose, glucose, xylans, and arabinose [13]. The hypocholesterolemic effects of dietary hull-less barley p-glucan (HBG) on cholesterol metabolism are reducing the concentration of plasma LDL cholesterol by promoting the excretion of fecal lipids and regulating the activities of HMG-CoA reductase and CYP7A1 in hypercholesterolemic hamsters [14].

In addition, other cereal's bran such as wheat, rice, and oat contains beta glucan that can also ameliorate lipid profile [15–17]. Heart, kidney and liver functions and histology are significantly affected with hypercholesterolemic [7, 17]. Feeding physically refined rice bran oil to hamsters can cause a reduction in plasma TC and LDL-C comparable or greater to that of canola oil-containing diets [18]. Peng et al. [19] reported that oat could act as adjuvant therapeutics for metabolic disorders via attenuating obesity and body fat and improving serum parameters with metabolic regulation and lipid clearance of liver. Tong et al. [20] stated that dietary oat oil improves hypercholesterolemia in rats fed a hypercholesterolemic diet, by promoting excretions of faecal lipids and bile acids.

This study was focused on testing the hypolipidemic activity of two doses of barley bran in improving the lipid profile and protecting hypercholesterolemic male rats against the fatty liver and heart.

## 2. Materials and Methods

### 2.1. Plant Materials

Barley bran was purchased from a local cereal mill in Jeddah, Saudi Arabia.

### 2.2. Basal Lipid Rich Diet

The basal diet consisted of the following: 16% casein, 10% corn oil, 4% N.N cellulose, 4% salt mixture, 1% vitamin mixture, 0.2% choline chloride, 0.2% DL. methionine, and 64.5% corn starch. The barley bran was added in a ratio of 5% or 10% subtracted from the corn starch percentage.

### 2.3. Animals and Housing Conditions

Twenty-four male albino rats (*Rattus norvegicus*) of East China Origin weighing 180–200 g were obtained from the Faculty of Pharmacy, King Abdulaziz University, Jeddah, Saudi Arabia. All the animal experiments were carried out under protocols approved by the Institutional Animal House of the University of King Abdulaziz at Jeddah, Saudi Arabia. Animals were housed six per polycarbonate cage. Cages, bedding, and glass water bottles (equipped with stainless steel sipper tubes) were replaced twice per week. The stainless steel feed containers were changed once per week.

### 2.4. Experiment Design

The animals were fed a basal lipid rich diet and kept under observation for 2 weeks before the start of the experiment to exclude any undercurrent infection. The test animals were divided randomly into four groups as follows: the first group (G1) is untreated control group and was fed basal lipid rich diet, the second group (G2) was fed 2% cholesterol in the lipid rich diet to induce hypercholesterolemia [21] and considered as the positive control group, the third group (G3) was fed 2% cholesterol and cotreated with 5% barley bran for 8 weeks, and the fourth group (G4) was fed 2% cholesterol and cotreated with 10% barley bran seed powder for 8 weeks. The experiment was conducted for 8 weeks as an adequate period to induce hypercholesterolemia [22].

### 2.5. Dissection

At the end of the experiment and after collection of blood, anaesthetized animals were sacrificed by cervical dislocation. The abdomen was dissected and the heart, the two kidneys, the two testes, and the liver were rapidly excised and weighed. A piece of the liver was saved in ice-cold for antioxidant enzymes and lipid peroxide estimation in tissue homogenate. The rest of the liver, the heart, and one kidney were saved in saline for histopathological investigations.

### 2.6. Physiological Evaluation

The following physiological parameters were estimated:

- daily water consumption,
- daily food intake,
- daily body weight gain (BWG),
- percentage of body weight gain (BWG%),
- food efficiency ratio (FER),
- percentage of food efficiency ratio (FER%),
- organs weight,
- relative organs weight.

### 2.7. Biochemical Tests

At the end of the experiment, animals were fasted 14–16 hours after their last feeding and blood samples were collected from the heart of dimethyl-ether preanaesthetized rats in plain tubes for biochemical analyses. Blood serum was obtained by centrifugation at 1000 rpm for 10 min at room temperature and then stored at −20°C until analysis was performed.

### 2.8. Preparation of Liver Tissue Homogenate

A piece of the liver tissue was dissected out, rinsed with ice-cold saline solution, and then homogenized in 0.1 M Tris–HCl buffer (pH 7.4) with a Teflon homogenizer at 4°C. The homogenate was centrifuged at 13000 ×g to remove the debris, and then the supernatant was used for estimation of the antioxidant enzymes and lipid peroxide.

### 2.9. Serum Lipids

Serum total cholesterol (STC), serum triglyceride (STG), and serum high density lipoprotein cholesterol were estimated using Spinreact kit (Spain) according to the instruction of the supplier. The value of serum low density lipoprotein cholesterol (S.LDLc) and serum very low density lipoproteins cholesterol (VLDLc) were calculated as follows:

- S.LDL-C = S.TC − (HDL-C + S.TG∖5),
- S.VLDL = S.TC − (S.LDL + S.HDL).

### 2.10. Lipid Peroxide

Lipid peroxide was estimated by determination of malondialdehyde (MDA) in the serum and in the liver tissue homogenate using Biodiagnostic Chemical Company kit (Egypt) according to the instructions of the suppliers.

### 2.11. Antioxidants Enzymes Estimation

Catalase, glutathione reduced (GSH), glutathione reductase (GR), and superoxide dismutase (SOD) were estimated in the serum and in liver tissue homogenate using the specified kits from Biodiagnostic Chemical Company (Egypt) according to the instructions of the suppliers.

### 2.12. Liver Enzymes

Serum alanine aminotransferase (ALT) was estimated using Human Kit (Germany), serum aspartate transaminase (AST) was estimated using Swemed diagnostics (India), serum alkaline phosphatase (ALP) was estimated using Human Kit (Germany), and serum Gamma-glutamyl transferase (GGT) was estimated using Abbott Diagnostics kit (USA). Estimation was done according to the instruction of the supplier.

### 2.13. Kidney Function Tests

Serum uric acid was estimated using Spinreact kit (Spain) according to the instruction of the supplier. Serum creatinine and serum urea were estimated using Biomerieux kit (France) according to the instruction of the supplier.

### 2.14. Estimation of Lactate Dehydrogenase

Lactate dehydrogenase was estimated using Human Kit (Germany) according to the instruction of the supplier.

### 2.15. Estimation of Creatine Kinase-MB

Creatine kinase-MB was estimated using Oxis International Inc. (USA) according to the instruction of the supplier.

### 2.16. Quantification of Bilirubin

Total bilirubin was estimated using Human kit (Germany) according to the instruction of the supplier.

### 2.17. Quantification of Total Protein

Total protein was quantified using Human kit (Germany) according to the instruction of the supplier.

### 2.18. Estimation of Albumins

Albumins were estimated using Sigma-Aldrich (USA) according to the instruction of the supplier.

### 2.19. Estimation of Globulins

Globulins were estimated using Human kit (Germany) according to the instruction of the supplier.

### 2.20. Albumins/Globulins Ratio

Albumins/globulins ratio was calculated.

### 2.21. Glycated Hemoglobin

Glycated hemoglobin (HbA_1c_) was estimated using Glycohemoglobin Reagent Set from Pointe Scientific Inc. (USA) according to the instructions of the suppliers.

### 2.22. Fasting Blood Sugar Determination

Fasting blood sugar determination was estimated using Glucose kit from Human (Germany) according to the instructions of the supplier.

### 2.23. Histopathological Investigations

The liver, heart, and one kidney were washed in sterile saline and fixed in 10% neutral formalin for histopathological studies. Organs were then dehydrated in gradual ethanol (50–99%), cleared in xylene, and embedded in paraffin. Sections were prepared and then stained with hematoxylin and eosin (H&S) dye for microscopic investigation according to [23]. The stained sections were examined and photographed under a light microscope.

### 2.24. Statistical Analysis

Values were analyzed using SPSS program to calculate the *t*-test and the mean ± SD and then analyzed using one way analysis of variance (ANOVA) using Duncan's Multiple Range Test [24].

## 3. Results

### 3.1. Organs Weight

Table 1 shows the effect of barley bran treatment on the weight of heart, liver, right kidney, left kidney, right testis, and left testis in hypercholesterolemic male rats. The mean value of most organs (heart, liver, right kidney, right testis, and left testis) was increased as a result of feeding rats of G2 fat rich diet and 2% cholesterol for 8 weeks, compared with the negative control. The concurrent supplementation with the two doses of barley bran in G3 and G4 significantly decreased all organs' weight compared with the positive control group.

### 3.2. Relative Organs Weight

Table 2 shows the effect of barley bran treatment on the relative weight of heart, liver, right kidney, left kidney, right testis, and left testis in hypercholesterolemic male rats. The mean relative value of all organs was increased as a result of feeding rats of G2 on fat rich diet and 2% cholesterol for 8 weeks, compared with the negative control. The concurrent supplementation with the two doses of barley bran in G3 and G4 significantly decreased all organs' relative weights compared with the positive control group.

### 3.3. Physiological Parameters

Table 3 shows the effect of barley bran supplementation for 8 weeks on physiological evaluations in hypercholesterolemic male rats. In G1, G2, and G3, the daily water consumption was nonsignificantly affected, whereas, in G4 (fed on 10% barley bran), it was significantly (at *P* < 0.05) increased compared with the negative control group. The daily food intake was not affected either by hypercholesterolemia or the concurrent supplementation with barley bran for 8 weeks, while the body weight gain was high significantly (at *P* < 0.001) increased in all groups. In addition, the percentage of BWG was significantly (at *P* < 0.05) increased with induction of hypercholesterolemia in G2, G3, and G4 compared with the negative control in G1. The food intake was not affected either by hypercholesterolemia or the concurrent supplementation with barley bran. The food efficiency and the food efficiency ratio were high significantly (at *P* < 0.001) increased in all groups. In addition, the percentage of BWG was significantly (at *P* < 0.05) increased under hypercholesterolemic conditions in G2 compared with the negative control in G1. The concurrent supplementation with barley bran for 8 weeks in G3 and G4 significantly decreased the FER and FER%.

### 3.4. Lipid Profile

Table 4 shows the effect of barley bran supplementation for 8 weeks on hypercholesterolemic male rats. The oral administration of 2% cholesterol to the rats of group (2) for 8 weeks has significantly (at *P* < 0.001) increased serum total cholesterol, serum triglycerides, serum low density lipoproteins, and serum very low density lipoproteins and decreased serum high density lipoproteins. The concurrent supplementation with 5% of barley bran to the hypercholesterolemic rats in G3 or 10% in G4 significantly (at *P* < 0.001) ameliorated all lipids parameters by decreasing serum total cholesterol, serum triglycerides, serum low density lipoproteins, and serum very low density lipoproteins and increasing serum high density lipoproteins. The higher dose of barley bran in G4 (10%) ameliorated lipid parameters more than the lower one in G3 in.

### 3.5. Serum Lipid Peroxide and Serum Antioxidants Enzymes

Table 5 shows the effect of barley bran supplementation for 8 weeks on serum lipid peroxide and antioxidants enzymes in hypercholesterolemic male rats. The oral administration of 2% cholesterol to the rats of group (2) for 8 weeks significantly (at *P* < 0.001) increased serum lipid peroxide and decreased serum catalase, SOD, GSH, and GR. The concurrent supplementation with 5% of barley bran in G3 and 10% of barley bran in G4 significantly (at *P* < 0.001) decreased serum lipid peroxide and increased serum catalase, SOD, GSH, and GR in the hypercholesterolemic rats. The higher dose of barley bran in G4 was more efficient in ameliorating lipid profile than the lower one in G3.

### 3.6. Lipid Peroxide and Antioxidants Enzymes in Liver Tissue Homogenate

The effect of barley bran supplementation for 8 weeks on lipid peroxide and antioxidants enzymes in liver tissue homogenate of hypercholesterolemic male rats is shown in Table 6. The oral administration of 2% cholesterol to the rats of group (2) for 8 weeks significantly (at *P* < 0.001) increased the lipid peroxide and decreased the catalase, SOD, GSH, and GR in the liver tissue homogenate. The concurrent supplementation of the hypercholesterolemic rats with 5% of barley bran in G3 and 10% of barley bran in G4 significantly (at *P* < 0.001) decreased the lipid peroxide and increased the catalase, SOD, GSH, and GR in the liver tissue homogenate. The higher dose of barley bran in G4 was more efficient in increasing antioxidant enzymes and decreasing lipid peroxide than the lower one in G3.

### 3.7. Liver Enzymes

In the positive control group (G2), liver enzymes (alanine aminotransferase, aspartate aminotransferase, alkaline phosphatase, and Gamma-glutamyl transferase) were significantly (at *P* < 0.001) increased as a result of cholesterol supplementation for 8 weeks as shown in Table 7. The concurrent supplementation with 5% of barley bran in G3 and 10% of barley bran in G4 to the hypercholesterolemic male rats significantly (at *P* < 0.001) decreased all liver enzymes under study. The higher dose of barley bran in G4 ameliorated the liver enzymes more than the lower one in G3.

### 3.8. Kidney Functions

Table 7 shows also that the mean value of kidney functions parameters (uric acid, creatinine, blood urea nitrogen) of the hypercholesterolemic rats in G2 was no significantly higher than that of the negative control group. The concurrent barley bran supplementation in G3 and G4 nonsignificantly decreased and ameliorated these kidney function parameters.

### 3.9. Lactate Dehydrogenase

The mean value of serum lactate dehydrogenase activity was significantly (at *P* < 0.001) increased in the positive control group (G2), as a result of cholesterol administration for 8 weeks as shown in Table 7. The concurrent supplementation with 5% of barley bran to the hypercholesterolemic male rats in G3 and 10% of barley bran in G4 for 8 weeks significantly (at *P* < 0.001) decreased lactate dehydrogenase in the serum of hypercholesterolemic rats under study. The higher dose of barley bran in G4 decreased lactate dehydrogenase more than the lower one in G3.

### 3.10. Creatine Kinase-MB

The serum creatine kinase-MB level was nonsignificantly increased as a result of induced hypercholesterolemia in G2 as shown also in Table 7. The concurrent supplementation of the two doses of barley bran in G3 and G4 nonsignificantly decreased the creatine kinase-MB to its normal levels in G1 as shown in Table 7.

### 3.11. Total Bilirubin

Table 8 shows also that the level of total bilirubin (the direct bilirubin and the indirect bilirubin) was not affected either by induced hypercholesterolemia in G2 or treating with the two doses of barley bran in G3 and G4.

### 3.12. Serum Proteins

Total protein (albumin, globulin, and their A/G ratio) were not affected with hypercholesterolemia as a result of cholesterol supplementation for 8 weeks in G2 or with the concurrent administration of the low dose of barley bran in G3 or the higher dose in G4, as shown in Table 8.

### 3.13. Fasting Blood Sugar

Fasting serum glucose level was also nonsignificantly affected by hypercholesterolemia in G2 or the concurrent treatment with barley bran in G3 and G4 as shown in Table 8.

### 3.14. Glycated Hemoglobin

The level of glycated hemoglobin was also not affected either by induced hypercholesterolemia in G2 or with the concurrent treatment with the two doses of barley bran in G3 and G4 as shown in Table 8.

### 3.15. Histopathology of the Heart

**Figure 1:**
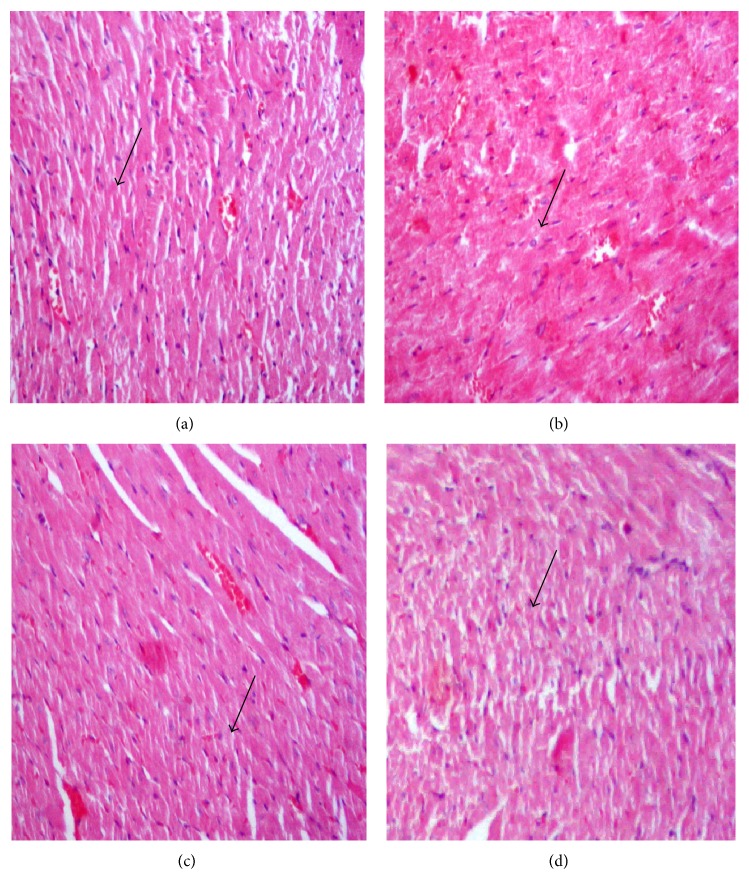
Histopathology of heart. (a) Normal cardiac tissues and normal muscle fibers (arrow) of negative control group fed basal diet, (b) cardiac tissues of positive control group fed 2% cholesterol for 8 weeks showing hyalinization of cardiac cells and damaged muscle fibers (arrow), (c) cardiac tissues of G3 that was fed 2% cholesterol and cosupplemented with 5% barley bran for 8 weeks showing restored normal cardiac tissues and muscle fibers (arrow), and (d) cardiac tissues of G3 that was fed 2% cholesterol and cosupplemented with 10% barley bran for 8 weeks showing restored normal structure (arrow) (H&E, ×400).

Figure 1 shows histology of the heart of the rats under study.  Figure 1(a) shows cardiac tissues of the negative control group rats with normal cardiac muscles.  Figure 1(b) shows cardiac tissues of hypercholesterolemic rats fed 2% cholesterol for 8 weeks in G2 showing fatty heart with increased hyalinization, cardiac muscles damage, and necrosis of muscle fibers.  Figure 1(c) shows cardiac tissues of hypercholesterolemic rats of the third group (G3) that was cosupplemented with 5% barley bran for 8 weeks showing minimal cardiac muscles damage and nearly restored normal tissues appearance.  Figure 1(d) shows cardiac tissues of hypercholesterolemic rats of the fourth group (G4) that was cosupplemented with 10% barley bran for 8 weeks showing minimal cardiac muscles damage and nearly restored normal tissues appearance.

### 3.16. Histopathology of the Liver

**Figure 2:**
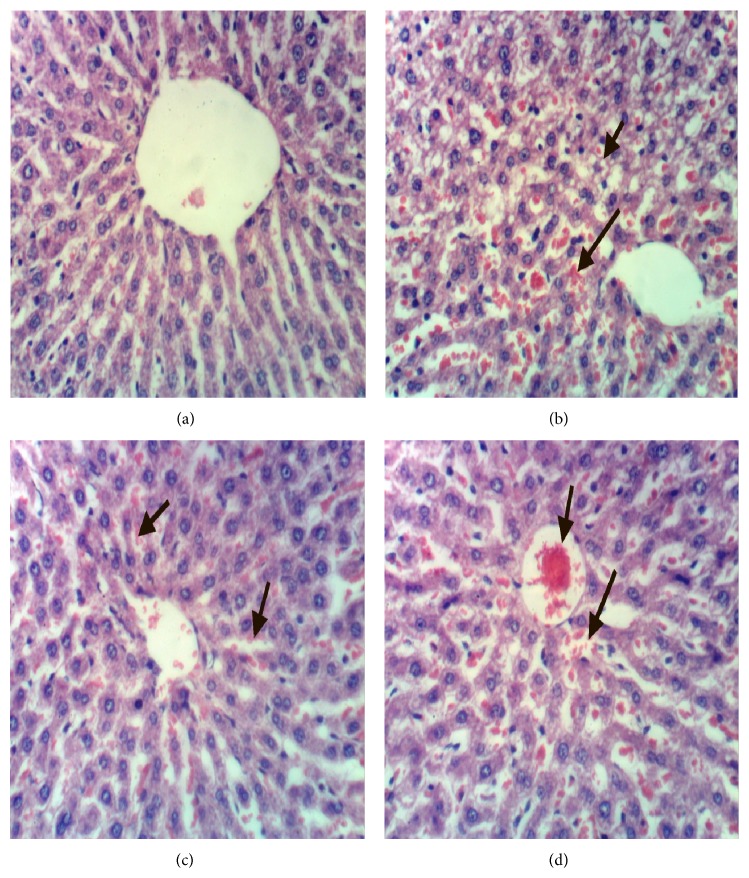
(a) Liver of rat from group 1 showing the normal histological structure of hepatic lobule, (b) liver of rat from group 2 showing steatosis of hepatocytes and focal hepatic necrosis associated with inflammatory cells infiltration, (c) liver of rat from group 3 showing slight congestion of hepatic sinusoids, and (d) liver of rat from group 4 showing congestion of central vein and hepatic sinusoids (H&E ×400).

The histology of liver is shown in Figure 2.  Figure 2(a) shows the hepatic tissues of the negative control group with normal hepatic strands of cells, blood sinusoids, and normal histological structure of hepatic lobule.  Figure 2(b) shows the hepatic tissues of hypercholesterolemic rats of the positive control group fed 2% cholesterol in fat rich diet for 8 weeks showing fatty hepatocytes and congestion of hepatic sinusoids, disrupted cells, disrupted hepatic strands, vacuolated cytoplasm, and necrosis. The hepatic tissues of hypercholesterolemic rats of the third group treated with 5% barley bran for 8 weeks showing nearly restored normal appearance of the hepatic strands with well-defined hepatic cords containing polyhedral hepatocytes and normal round nuclei (Figure 2(c)).  Figure 2(d) shows hepatic tissues of hypercholesterolemic rats treated with 10% barley bran for 8 weeks with restored normal hepatic appearance and normal hepatic strands.

### 3.17. Histopathology of the Kidney

**Figure 3:**
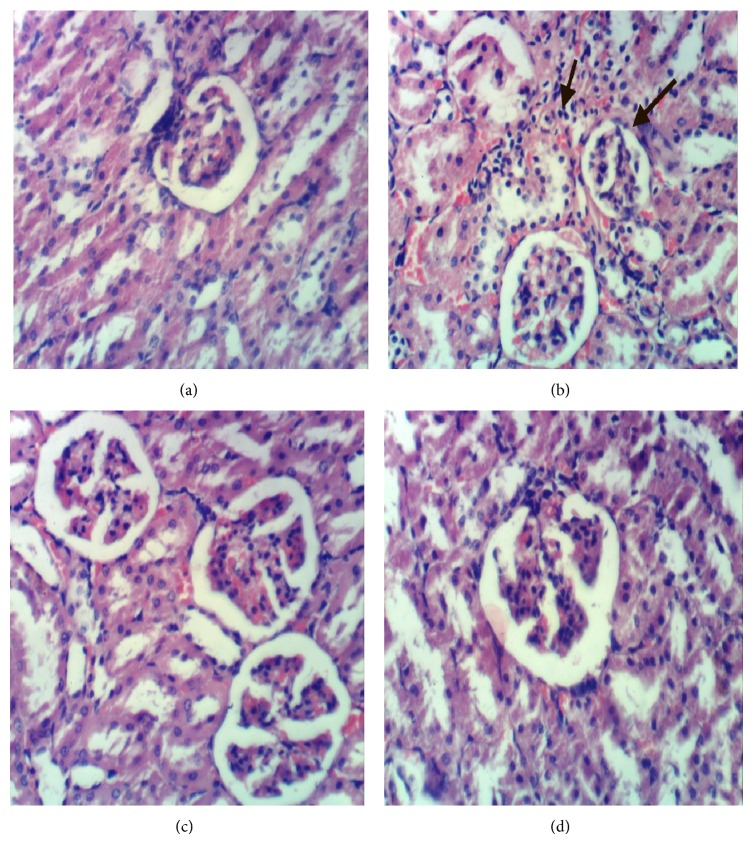
(a) Kidney of rat from the negative control group G1 showing normal histological structure of renal parenchyma and glomeruli, (b) kidney of hypercholesterolemic rat from G2 showing atrophy of glomerular tuft, (c) kidney of rat from G3 showing no histopathological changes, and (d) kidney of rat from group 4 showing no histopathological changes (H&E ×400).

Figure 3 shows the histopathology of kidney of the negative control group with normal histological structure of renal parenchyma, blood vessels and interstitium with no histopathological changes, normal glomeruli with normal structure and pattern, normal renal tubuli in living epithelium and normal interstitial tissue with normal in composition, and normal blood vessels as shown in Figure 3(a).  Figure 3(b) shows the renal tissues of the positive control group that was fed fat rich diet with 2% cholesterol for 8 weeks, showing shrinkage of glomerular tuft, congestion of glomerular capillaries, mild tubular atrophy, interstitial edema with formation of scattered intratubular granules, and atrophy of glomerular tuft. Kidney of the third group that was fed 2% cholesterol diet and cosupplemented with 5% barley bran for 8 weeks shows restored kidney tissue with normal architecture and normal glomeruli with normal structure and pattern with no histopathological changes as shown in Figure 3(c). Kidney of the fourth group that was fed 2% cholesterol diet and cosupplemented with 10% barley bran for 8 weeks shows restored kidney tissue with normal architecture and normal glomeruli with normal structure and pattern with histopathological changes as shown in Figure 3(d).

## 4. Discussion

The hypolipidemic activity of two doses of barley bran seeds powder and their efficiency in protecting the heart in male albino rats were studied using biochemical and histological investigations. Diet is a key modifiable risk factor in the prevention and risk reduction of coronary heart disease [7]. Dietary wheat bran arabinoxylans reduced the plasma total cholesterol and LDL-cholesterol concentrations by promoting the excretion of fecal lipids, regulating the activities of HMG-CoA reductase and CYP7A1 and increasing colonic SCFAs in hamsters [20].

In the current study, feeding rats on 2% cholesterol in the diet for 8 weeks increased the serum total cholesterol and induced hypercholesterolemia in the positive control group. This result is consistent with previous studies [17, 21, 25]. The concurrent oral administration of 5% and 10% barley bran to the hypercholesterolemic rats of G3 and G4, respectively, for 8 weeks significantly improved the serum lipid profile parameters. It decreased the total cholesterol, triglycerides, low density lipoproteins, and very low density lipoproteins and increased the useful cholesterol (high density lipoproteins). This result agrees with previous investigations [15, 17, 26]. Dietary fiber is used more frequently in the treatment of diabetes, hypercholesterolemia, and diverticulitis through its effect on gastrointestinal function and reduction of low-density lipoprotein (LDL) cholesterol in hypercholesterolemic animals [17, 26–28].

Hypercholesterolemia occurs in conjunction with other metabolic risk factors including glucose intolerance, obesity, diabetes, metabolic syndromes, and oxidation of the lipid core of low-density lipoproteins that leads to a change in the lipoprotein conformation [8, 21]. The increase of lipid peroxide and the decrease of antioxidant enzymes under study as a result of hypercholesterolemia in serum and liver tissue homogenate of the positive control group are consistent with other studies [8, 17, 21]. The concurrent supplementation with barley bran to the hyperlipidemic rats in G3 and G4 greatly ameliorated the lipid peroxide and antioxidant levels [17, 29]. Barley bran phenolic acids such as ferulic acid may scavenge free-radical oxygen species both* in vitro* and* in vivo*; however, it has lower antioxidant activity* in vivo* compared with* in vitro* that was higher [8].

Furthermore, liver function parameters (serum alanine aminotransferase, serum aspartate transaminase, serum Gamma-glutamyl transferase, and serum alkaline phosphatase) were also significantly increased under induced hyper lipidemic condition in male rats. This result is consistent with that of Mahfouz and Kummerow [30] and El Rabey et al. [17]. The concurrent supplementation with the two doses of barley bran to the hypercholesterolemic rats in G3 and G4 has significantly ameliorated the liver enzymes under study and nearly restored them to the normal conditions. This result is consistent with previous investigations [8, 17, 21].

In contrast, kidney functions, glycated hemoglobin, and fasting sugar were nonsignificantly increased with hypercholesterolemia in G2. The concurrent barley bran supplementation in G3 and G4 ameliorated these parameters. This result which is consistent with previous investigations revealed that dietary fibers improve kidney functions [17, 21, 31].

The heart function enzymes, lactate dehydrogenase and creatine kinase-MB, were also increased under induced hyperlipidemic condition in the G2 rats. This result is consistent with that of Rathod et al. [21]. Lactate dehydrogenase and creatine kinase-MB are released during heart tissue damage resulted from hypercholesterolemia. The concurrent supplementation with the two doses of barley bran to the hypercholesterolemic rats in G3 and G4 has significantly ameliorated the heart enzymes under study and nearly restored them to the normal conditions. This result is consistent with previous investigations [17, 21, 29].

In addition, total bilirubin, total protein, albumins, globulins, and albumins/globulins ratio were not affected with either hypercholesterolemia or the concurrent supplementation with barley bran, in the current study. On the other hand, water consumption and food intake were not affected by hypercholesterolemia or treating with barley bran seeds powder, whereas body weight gain and food efficiency ratio were significantly increased by hypercholesterolemia and decreased by treatment with barley bran in for 8 weeks in G3 and G4.

The hepatic and renal tissues were significantly affected in hypercholesterolemic rats of G2 as a result of the 2% cholesterol supplementation. They showed fatty hepatocytes and congestion of hepatic sinusoids, disrupted cells, disrupted hepatic strands, vacuolated cytoplasm, and necrosis. This result which is consistent with other studies showed a correlation between hypercholesterolemia and pathological alteration of vital organs [17, 32, 33]. The concurrent supplementation with barley bran in G3 and G4 has significantly improved the liver tissues and nearly restored them to their normal state [17, 34].

It could be concluded that the two low doses of barley bran under study succeeded in lowering hyperlipidemia induced by cholesterol feeding for 8 weeks in male rats by lowering TC, TG, LDLc, and VLDLC and increasing HDLc levels in the treated groups. Barley bran supplementation has also improved liver enzymes and heart enzymes and nearly restored tissues of the kidney and liver to their normal structure. The high dose of barley bran in G4 was more effective than the lower dose in G3.

## Figures and Tables

**Table 1:** Effect of barley bran supplementation for 8 weeks on organs weight in hypercholesterolemic rats.

Organs weight g	Statistics	G1	G2	G3	G4
		−ve control	+ve control	5% barley bran	10% barley bran
Heart	Mean ± SE	7.41 ± 0.30^a^	8.51 ± 0.30^b^	6.91 ± 0.22^c^	7.21 ± 0.27^b^
	LSD 0.05 = 0.103				
	*t*-test		−4.19^***^	4.39^***^	2.58^*^

Liver	Mean ± SE	0.56 ± 0.06^a^	0.65 ± 0.07^b^	0.66 ± 0.06^a^	0.63 ± 0.03^a^
	LSD 0.05 = 0.579				
	*t*-test		−0.88^*^	−0.15^NS^	0.19^NS^

Right kidney	Mean ± SE	0.60 ± 0.05^a^	0.71 ± 0.01^b^	0.66 ± 0.04^a^	0.66 ± 0.02^a^
	LSD 0.05 = 0.077				
	*t*-test		−2.15^*^	1.16^NS^	2.23^NS^

Left kidney	Mean ± SE	0.78 ± 0.03^a^	0.66 ± 0.02^b^	0.63 ± 0.03^a^	0.66 ± 0.04^a^
	LSD 0.05 = 1.103				
	*t*-test		2.90^*^	1.00^NS^	0.00^NS^

Right testes	Mean ± SE	1.13 ± 0.07^a^	1.31 ± 0.06^d^	1.13 ± 0.06^c^	1.08 ± 0.03^b^
	LSD 0.05 = 0.106				
	*t*-test		−1.43^**^	4.56^**^	3.07^**^

Left testes	Mean ± SE	1.16 ± 0.05^a^	1.26 ± 0.03^b^	1.13 ± 0.07^c^	1.16 ± 0.04^b^
	LSD 0.05 = 0.330				
	*t*-test		−1.16^*^	1.86^*^	1.22^*^

Data are represented as mean ± SE. *t*-test values; ^***^ *P* < 0.001. ANOVA analysis: within each row, means with different superscript (a, b, c, or d) are significantly different at *P* < 0.05, whereas means superscripts with the same letters mean that there is no significant difference at *P* < 0.05. LSD: least significant difference. ^*^Significant, ^**^highly significant and ^***^very high significant.

**Table 2:** Effect of barley bran on relative organs weight in hypercholesterolemic rats for 8 weeks.

Relative organ weight (ROW) %	Treatments	G1	G2	G3	G4
	statistics	−ve control	+ve control	5% barley bran	10% barley bran
Heart	Mean ± SE	0.26 ± 0.02^a^	0.40 ± 0.04^b^	0.30 ± 0.03^b^	0.28 ± 0.01^b^
	LSD 0.05 = 0.016				
	*t*-test		−2.71^*^	1.84^NS^	2.45^**^

Liver	Mean ± SE	3.41 ± 0.14^a^	5.36 ± 0.18^b^	3.13 ± 0.10^b^	3.22 ± 0.13^b^
	LSD 0.05 = 0.381				
	*t*-test		−13.55^***^	11.19^***^	7.76^***^

Right kidney	Mean ± SE	0.27 ± 0.02^a^	0.45 ± 0.01^c^	0.30 ± 0.01^b^	0.29 ± 0.01^b^
	LSD 0.05 = 0.034				
	*t*-test		−6.71^***^	7.75^***^	12.17^***^

Left kidney	Mean ± SE	0.36 ± 0.01^a^	0.42 ± 0.01^b^	0.28 ± 0.01^b^	0.29 ± 0.02^b^
	LSD 0.05 = 0.049				
	*t*-test		−2.79^*^	8.42^***^	5.07^***^

Right testes	Mean ± SE	0.52 ± 0.03^a^	0.82 ± 0.03^b^	0.51 ± 0.02^b^	0.48 ± 0.01^b^
	LSD 0.05 = 0.044				
	*t*-test		−4.44^***^	12.62^***^	7.88^***^

Left testes	Mean ± SE	0.53 ± .025^a^	0.79 ± 0.02^b^	0.51 ± 0.03^b^	0.51 ± 0.02^b^
	LSD 0.05 = 0.076				
	*t*-test		−5.77^***^	8.69^***^	6.70^***^

Data are represented as mean ± SE. *t*-test values; ^***^ *P* < 0.001. ANOVA analysis: within each row, means with different superscript (a, b, c, or d) are significantly different at *P* < 0.05, whereas means superscripts with the same letters mean that there is no significant difference at *P* < 0.05. LSD: least significant difference.^*^Significant, ^**^highly significant and ^***^very high significant.

**Table 3:** Effect of barley bran on food intake (FI) body weight gain (BWG) and food efficiency ratio (FER) in hypercholesterolemic rats for 8 weeks.

Biological evaluation parameters	Treatments	G1	G2	G3	G4
	statistics	−ve control	+ve control	5% barley bran	10% barley bran
Water consumption mL/day	Mean ± SE	30.29 ± 0.67^a^	30.55 ± 0.63^a^	31.50 ± 0.66^a^	32.66 ± 0.57^a^
	LSD 0.05 = 1.342				
	*t*-test		−0.32^NS^	−1.36^NS^	−3.45^***^

FI g/day	Mean ± SE	17.07 ± 0.19^a^	17.12 ± 0.20^a^	17.20 ± 0.21^a^	17.05 ± 0.21^a^
	LSD 0.05 = 0.341				
	*t*-test		−0.40^NS^	−0.46^NS^	0.58^NS^

BWG g/8 week	Mean ± SE	32.83 ± 1.85^a^	37.00 ± 1.06^b^	34.50 ± 0.88^c^	33.50 ± 3.47^b^
	LSD 0.05 = 6.330				
	*t*-test		−2.66^***^	4.83^***^	5.98^***^

BWG %	Mean ± SE	17.83 ± 1.08^a^	20.09 ± 6.50^b^	18.50 ± 0.53^c^	17.70 ± 2.04^d^
	LSD 0.05 = 3.780				
	*t*-test		−2.97^*^	2.78^**^	3.20^**^

FER g/day	Mean ± SE	0.031 ± 0.00^a^	0.026 ± 0.00^b^	0.033 ± 0.00^d^	0.032 ± 0.00^c^
	LSD 0.05 = 0.010				
	*t*-test		0.02^***^	−0.04^***^	−0.03^***^

FER %	Mean ± SE	3.199 ± 0.18^a^	3.80 ± 0.87^d^	3.37 ± 0.08^b^	3.27 ± 0.33^c^
	LSD 0.05 = 1.033				
	*t*-test		−1.04^***^	1.45^***^	1.17^***^

Data are represented as mean ± SE. *t*-test values; ^***^ *P* < 0.001. ANOVA analysis: within each row, means with different superscript (a, b, c, or d) are significantly different at *P* < 0.05, whereas means superscripts with the same letters mean that there is no significant difference at *P* < 0.05. LSD: least significant difference.^*^Significant, ^**^highly significant and ^***^very high significant.

**Table 4:** Effect of barley bran supplementation for 8 weeks on serum lipids of hypercholesterolemic male rats.

Parameters	Statistics	G1	G2	G3	G4
		−ve control	+ve control	5% barley bran	10% barley bran
S.TC mg%	Mean ± SE	132.67 ± 2.83^a^	292.83 ± 1.42^d^	238.83 ± 1.70^b^	200.83 ± 3.21^c^
	LSD 0.05 = 7.438				
	*t*-test		−41.22^***^	19.67^***^	23.00^***^

S.T.G mg/dL	Mean ± SE	95.50 ± 1.17^a^	275.33 ± 1.05^d^	206.83 ± 1.95^b^	125.67 ± 1.42^c^
	LSD 0.05 = 18.502				
	*t*-test		−83.00^***^	25.32^***^	79.12^***^

S.HDLc mg/dL	Mean ± SE	53.66 ± 1.30^a^	25.66 ± 0.84^a^	27.33 ± 0.21^c^	38.50 ± 0.92^b^
	LSD 0.05 = 2.855				
	*t*-test		13.66^***^	−1.746	−8.73^***^

S.LDLc mg/dL	Mean ± SE	59.90 ± 2.12^a^	212.10 ± 1.67^d^	169.60 ± 1.72^b^	137.20 ± 2.72^c^
	LSD 0.05 = 6.702				
	*t*-test		−46.37^***^	16.49^***^	22.81^***^

V.LDLc mg/dL	Mean ± SE	19.10 ± 0.23^a^	55.06 ± 0.21^d^	41.36 ± 0.39^b^	25.16 ± 0.29^c^
	LSD 0.05 = 0.916				
	*t*-test		−83.00^***^	25.32^***^	78.25^***^

Data are represented as mean ± SE. *t*-test values; ^***^ *P* < 0.001. ANOVA analysis: within each row, means with different superscript (a, b, c, or d) are significantly different at *P* < 0.05, whereas means superscripts with the same letters mean that there is no significant difference at *P* < 0.05. LSD: least significant difference.

**Table 5:** Effect of barley bran supplementation for 8 weeks on serum antioxidants enzymes in hypercholesterolemic rats.

Parameters	Statistics	G1	G2	G3	G4
Serum		−ve control	+ve control	5% barley bran	10% barley bran
MDA nmol/mL Serum	Mean ± SE	2.06 ± 0.08^a^	10.68 ± 0.21^d^	7.33 ± 0.20^b^	4.63 ± 0.20^c^
	LSD 0.05 = 0.624				
	*t*-test		−38.22^***^	12.35^***^	15.39^***^

Catalase (CAT) U/mL	Mean ± SE	4.64 ± 0.11^a^	0.58 ± 0.03^d^	1.33 ± 0.08^c^	2.56 ± 0.09^b^
	LSD 0.05 = 0.249				
	*t*-test		33.03^***^	−10.50^***^	−26.18^***^

Superoxide dismutase (SOD) U/mL	Mean ± SE	851.38 ± 2.42^a^	385.08 ± 2.05^d^	450.55 ± 7.00^c^	616.90 ± 2.37^b^
	LSD 0.05 = 76.231				
	*t*-test		125.99^***^	−10.48^***^	−98.29^***^

Glutathione reduced (GSH) U/mL	Mean ± SE	23.91 ± 1.02^a^	1.54 ± 0.03^d^	7.16 ± 0.18^c^	12.11 ± 20.86^b^
	LSD 0.05 = 1.492				
	*t*-test		21.78^***^	−28.22^***^	−43.09^***^

Glutathione reductase (GR) U/mL	Mean ± SE	617.11 ± 20.86^a^	216.12 ± 4.24^d^	341.32 ± 46.43^c^	470.43 ± 15.22^b^
	LSD 0.05 = 86.013				
	*t*-test		179.77^***^	−26.97^***^	−150.24^***^

Data are represented as mean ± SE. *t*-test values; ^***^ *P* < 0.001. ANOVA analysis: within each row, means with different superscript (a, b, c, or d) are significantly different at *P* < 0.05, whereas means superscripts with the same letters mean that there is no significant difference at *P* < 0.05. LSD: least significant difference.

**Table 6:** Effect of barley bran supplementation for 8 weeks on liver tissue lipid peroxidation and antioxidants enzymes in hypercholesterolemic male rats.

Parameters	Statistics	G1	G2	G3	G4
liver tissue		−ve control	+ve control	5% barley bran	10% barley bran
MDA nmol/g. liver tissue	Mean ± SE	3.10 ± 0.09^a^	13.01 ± 0.20^d^	9.21 ± 0.23^b^	5.41 ± 0.09^c^
	LSD 0.05 = 0.475				
	*t*-test		−44.76^***^	16.45^***^	30.52^***^

Catalase (CAT) U/g. liver tissue	Mean ± SE	25.93 ± 0.90^a^	4.01 ± 0.21^d^	9.46 ± 0.33^c^	14.45 ± 0.47^b^
	LSD 0.05 = 1.549				
	*t*-test		24.85^***^	−10.34^***^	−16.86^***^

Superoxide dismutase (SOD) U/g. liver tissue	Mean ± SE	111.68 ± 3.04^a^	64.46 ± 4.01^d^	76.19 ± 3.30^c^	95.86 ± 29.81^b^
	LSD 0.05 = 8.133				
	*t*-test		138.76^***^	−28.57^***^	−50.11^***^

Glutathione reduced (GSH) U/g. liver tissue	Mean ± SE	66.88 ± 1.78^a^	26.81 ± 1.08^d^	34.28 ± 0.59^c^	46.71 ± 0.47^b^
	LSD 0.05 = 3.714				
	*t*-test		15.70^***^	−7.24^***^	−14.06^***^

Glutathione reductase (GR) U/g. liver tissue	Mean ± SE	780.47 ± 13.64^a^	342.90 ± 8.48^d^	389.22 ± 16.67^c^	549.27 ± 22.67^b^
	LSD 0.05 = 51.271				
	*t*-test		205.72^***^	−24.67^***^	−78.91^***^

Data are represented as mean ± SE. *t*-test values; ^***^ *P* < 0.001. ANOVA analysis: within each row, means with different superscript (a, b, c, or d) are significantly different at *P* < 0.05, whereas means superscripts with the same letters mean that there is no significant difference at *P* < 0.05. LSD: least significant difference.

**Table 7:** Effect of barley bran supplementation for 8 weeks on serum liver, kidney function, serum lactate dehydrogenase, and creatine kinase-MB in hypercholesterolemic male rats.

Parameters	Statistics	G1	G2	G3	G4
		−ve control	+ve control	5% barley bran	10% barley bran
ALT U/dL	Mean ± SE	34.40 ± 1.60^a^	88.36 ± 1.72^d^	78.78 ± 1.02^b^	57.20 ± 0.51^c^
	LSD 0.05 = 4.001				
	*t*-test		−18.89^***^	12.32^***^	17.77^***^

AST U/dL	Mean ± SE	33.21 ± 1.30^a^	78.65 ± 0.67^d^	63.90 ± 1.09^b^	51.25 ± 0.54^c^
	LSD 0.05 = 2.536				
	*t*-test		−40.43^***^	11.69^***^	37.87^***^

ALP U/dL	Mean ± SE	118.33 ± 4.86^a^	222.67 ± 4.27^d^	186.67 ± 1.99^b^	160.33 ± 1.38^c^
	LSD 0.05 = 10.783				
	*t*-test		−13.71^***^	7.67^***^	14.46^***^

GGT U/dL	Mean ± SE	22.50 ± 1.47^a^	68.50 ± 0.76^d^	59.16 ± 0.94^b^	44.66 ± 1.05^c^
	LSD 0.05 = 3.079				
	*t*-test		−23.39^***^	8.60^***^	19.94^***^

Uric acid mg/dL	Mean ± SE	2.35 ± 0.26^a^	2.63 ± 0.26^a^	2.48 ± 0.26^a^	2.51 ± 0.28^a^
	LSD 0.05 = 0.930				
	*t*-test		0.98^NS^	−0.27^NS^	−0.80^NS^

Creatinine mg/dL	Mean ± SE	0.54 ± 0.03^a^	0.56 ± 0.03^a^	0.55 ± 0.03^a^	0.55 ± 0.03^a^
	LSD 0.05 = 0.079				
	*t*-test		−0.22^NS^	0.00^NS^	−0.08^NS^

Blood urea nitrogen mg/dL	Mean ± SE	23.63 ± 1.72^a^	23.93 ± 1.64^a^	22.23 ± 1.69^a^	23.31 ± 1.53^a^
	LSD 0.05 = 5.461				
	*t*-test		−0.14^NS^	0.45^NS^	0.10^NS^

Lactate dehydrogenase LDH U/L	Mean ± SE	178.00 ± 5.21^d^	455.67 ± 8.80^a^	402.00 ± 4.28^b^	294.17 ± 4.51^c^
	LSD 0.05 = 18.601				
	*t*-test		−20.76^***^	6.69^***^	23.46^***^

Creatine kinase-MB (CK-MB) ug/mL (mcg/mL)	Mean ± SE	20.35 ± 1.07^a^	21.53 ± 1.00^a^	20.90 ± 1.17^a^	20.63 ± 0.98^a^
	LSD 0.05 = 3.357				
	*t*-test		−0.78^NS^	0.34^NS^	0.97^NS^

Data are represented as mean ± SE. *t*-test values; ^***^ *P* < 0.001. ANOVA analysis: within each row, means with different superscript (a, b, c, or d) are significantly different at *P* < 0.05, whereas means superscripts with the same letters mean that there is no significant difference at *P* < 0.05. LSD: least significant difference.

**Table 8:** Effect of barley bran supplementation for 8 weeks on bilirubin, protein, fasting blood sugar, and glycated hemoglobin in hypercholesterolemic rats.

Parameters	Statistics	G1	G2	G3	G4
Total bilirubin mg/dL	Mean ± SE	0.73 ± 0.06^a^	0.73 ± 0.06^a^	0.71 ± 0.06^a^	0.70 ± 0.06^a^
	LSD 0.05 = 8.197				
	*t*-test		0.00^NS^	0.19^NS^	0.37^NS^

Direct bilirubin mg/dL	Mean ± SE	0.20 ± 0.02^a^	0.21 ± 0.03^a^	0.18 ± 0.03^a^	0.20 ± 0.03^a^

Indirect bilirubin mg/dL	LSD 0.05 = 0.137	0.53 ± 0.04^a^	0.51 ± 0.04^a^	0.53 ± 0.04^a^	0.50 ± 0.03^a^
	*t*-test		0.41^NS^	−0.27^NS^	0.23^NS^
	LSD 0.05 = 0.137	0.53 ± 0.04^a^	0.51 ± 0.04^a^	0.53 ± 0.04^a^	0.50 ± 0.03^a^

Total protein g/dL	Mean ± SE	6.80 ± 0.21^a^	6.71 ± 0.23^a^	6.76 ± 0.19^a^	6.81 ± 0.20^a^
	LSD 0.05 = 0.060				
	*t*-test		0.29^NS^	−0.13^NS^	−0.29^NS^

Albumin g/dL	Mean ± SE	4.23 ± 0.13^a^	4.23 ± 0.11^a^	4.28 ± 0.15^a^	4.25 ± 0.13^a^
	LSD 0.05 = 0.376				
	*t*-test		0.00^NS^	−0.22^NS^	−0.10^NS^

Globulin g/L	Mean ± SE	2.56 ± 0.25^a^	2.48 ± 0.18^a^	2.48 ± 0.22^a^	2.56 ± 0.27^a^
	LSD 0.05 = 0.698				
	*t*-test		0.23^NS^	0.00^NS^	−0.22^NS^

A/G ratio g/L	Mean ± SE	1.76 ± 0.23^a^	1.77 ± 0.13^a^	1.82 ± 0.23^a^	1.79 ± 0.25^a^
	LSD 0.05 = 0.60				
	*t*-test		−0.03^NS^	−0.13^NS^	−0.04^NS^

Fasting blood sugar (FBS) mg/dL	Mean ± SE	113.17 ± 4.16^a^	115.50 ± 3.41^a^	112.17 ± 4.86^a^	112.33 ± 4.65^a^
	LSD 0.05 = 13.33				
	*t*-test		−0.34^NS^	0.49^NS^	0.46^NS^

Glycated hemoglobin A1c %	Mean ± SE	5.01 ± 0.10^a^	5.08 ± 0.09^a^	5.08 ± 0.06^a^	5.08 ± 0.08^a^
	LSD 0.05 = 0.268				
	*t*-test		−0.39^NS^	0.00^NS^	0.00^NS^

Data are represented as mean ± SE. *t*-test values; ^***^ *P* < 0.001. ANOVA analysis: within each row, means with different superscript (a, b, c, or d) are significantly different at *P* < 0.05, whereas means superscripts with the same letters mean that there is no significant difference at *P* < 0.05. LSD: least significant difference.

## Abbreviations

A1c: Glycated hemoglobin
ALP: Serum alkaline phosphatase
ALT: Serum alanine aminotransferase
AST: Serum aspartate aminotransferase
b.w.: Body weight
BUN: Blood urea nitrogen
Cat: Catalase
Cr: Creatinine
FB: Fasting blood sugar
GGT: Gamma-glutamyl transferase
G1: Negative control group
G2: Positive control group
G3: Hypercholesterolemic rats treated with 5% barley bran
G4: Hypercholesterolemic rats treated with 10% barley bran
GR: Glutathione reductase
GSH: Glutathione reduced
LDH: Lactate dehydrogenase
LDLc: Low density lipoprotein cholesterol
SOD: Activity of superoxide dismutase
TC: Total cholesterol
TG: Triglyceride
VLDLC: Very low density lipoproteins cholesterol.
